# Targeting bone marrow mechanosensation in myelofibrosis

**DOI:** 10.1002/hem3.46

**Published:** 2024-03-17

**Authors:** Francesca Vinchi

**Affiliations:** ^1^ Iron Research Laboratory Lindsley F. Kimball Research Institute, New York Blood Center New York New York USA; ^2^ Department of Pathology and Laboratory Medicine Weill Cornell Medicine New York New York USA

Primary myelofibrosis (PMF) is a chronic myeloproliferative neoplasm characterized by progressive accumulation of fibrosis in the bone marrow.^1^ PMF results from the neoplastic transformation of a pluripotent hematopoietic stem cell. PMF progeny cells stimulate bone marrow fibroblasts—which are not part of the neoplastic transformation—to produce excessive collagen. As scar tissue increases, bone marrow failure eventually occurs, with consequent anemia and thrombocytopenia.^1^

Mutations of the Janus kinase 2 (*JAK2*) gene are present in a high proportion of cases of PMF. JAK2 is a member of the class I type tyrosine kinase family of enzymes and is involved in signal transduction initiated by erythropoietin, thrombopoietin, and granulocyte colony‐stimulating factor receptors among other stimuli. Mutations of the thrombopoietin receptor gene (*MPL*) or the calreticulin (*CALR*) gene also may be the cause of PMF.

PMF hallmarks are megakaryocytosis, with atypical immature megakaryocytes and the accumulation of cross‐linked collagen and reticulin fibers in the bone marrow, which gradually alter the architecture and stiffness of the extracellular matrix.^1^ With the progression of the disease, patients with PMF often present with thrombocytopenia and high‐grade fibrosis, which are indicative of a more advanced disease state.

The mechanical properties of the extracellular matrix within the bone marrow microenvironment exert a crucial role in various aspects of megakaryocyte development, including lineage commitment, polyploidization, and proplatelet formation. A softer environment likely supports these differentiation and maturation processes. Indeed, megakaryocytes cultured on soft substrates exhibit enhanced maturation and platelet production compared to stiffer surfaces.^2^, ^3^ However, how this sensing occurs and the membrane mechanosensor that mediates it and plays a role in megakaryocytes have remained unknown so far. A recent study published in the *American Journal of Hematology* unveiled the involvement of the mechanosensor PIEZO1 in the regulation of megakaryocyte maturation and proplatelet biogenesis under physiological and pathological conditions.

Mechanosensitive ion channels are integral membrane proteins that play a crucial role in fast signaling during mechanosensory transduction processes in living cells. As molecular transducers of mechanical force, mechanosensitive channels are activated by mechanical stimuli exerted on cellular membranes, upon which they rapidly and efficiently convert these stimuli into electrical, osmotic, and/or chemical intracellular signals. PIEZO1 is a cation channel that senses and transduces externally applied mechanical forces acting on the lipid plasma membrane and influences cell fate and tissue homeostasis in multiple cell types and tissues. PIEZO1 has a role in different blood cells, including red blood cells, macrophages, and platelets.^4^, ^5^, ^6^, ^7^ In erythrocytes, PIEZO1 controls cell differentiation and hydration status following mechanical activation^4^, ^5^; in macrophages, it modulates macrophage polarization and stiffness sensing^6^; in platelets, PIEZO1 activation by shear stress regulates calcium flux and thrombotic activity.^7^

Abbonante et al.^8^ recently proved a key role for PIEZO1 in megakaryopoiesis by taking advantage of mice deficient of the *Piezo* genes in the megakaryocyte lineage. Mice lacking PIEZO in megakaryocytes showed an increased number of circulating platelets both at steady state and upon myeloregeneration, suggesting a role for PIEZO activation in restraining platelet production. Indeed, the use of PIEZO1 pharmacological agonists on mouse and human cells increased immature megakaryocytes and decreased mature ones, reducing proplatelet formation via impaired megakaryocyte maturation.

Since PMF is associated with a severely modified extracellular matrix in the bone marrow, mechanical sensing is likely altered in this pathological condition. Interestingly, megakaryocytes from *JAK2* ^V617F^ mice as well as megakaryocytes and platelets from PMF patients showed an increased expression of PIEZO1 at messenger RNA and protein levels. In particular, PIEZO1 expression was higher in platelets from patients showing overt compared to early PMF, being characteristic of an advanced fibrotic disease. Indeed, PIEZO expression increased when human megakaryocytes were grown in vitro into a stiff compared to a soft three‐dimensional matrix, indicating that, in PMF alterations of bone marrow, stiffness results in PIEZO1 overexpression (Figure 1). Finally, the negative correlation between megakaryocyte PIEZO1 expression and blood platelet count in patients with PMF confirmed a role for PIEZO1 as a negative regulator of megakaryocyte maturation and platelet production. Overall, these findings support a role for PIEZO1 overexpression in the pathological megakaryopoiesis and reduced platelet production observed in PMF. Interestingly, the use of PIEZO1 inhibitors promoted PMF megakaryocyte maturation and proplatelet formation in vitro. This underscores PIEZO1 inhibition as a potential therapeutic strategy to mitigate the impaired megakaryocyte development underlying PMF pathophysiology. Future studies will determine whether the increased PIEZO1 expression observed in platelets from patients with PMF might contribute to the increased thrombotic risk that is described in these patients.

**Figure 1:**
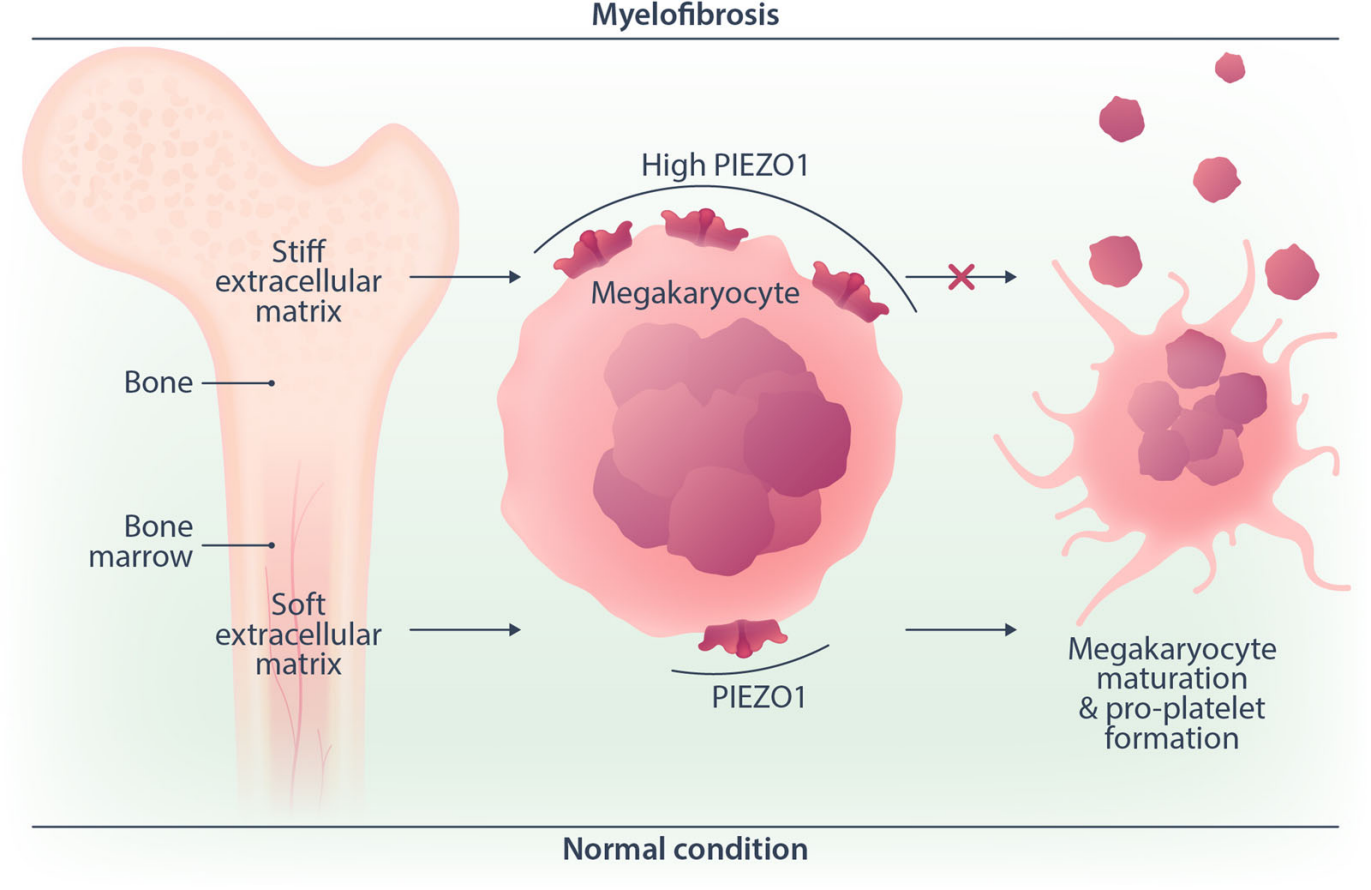
Role of PIEZO1 in megakaryocyte maturation and platelet formation under normal and pathological condition. The expression and activation of PIEZO1 are regulated by the stiffness of the extracellular matrix within the bone marrow microenvironment. Under physiologic conditions, a soft microenvironment allows a normal megakaryocyte maturation and platelet production, via moderate PIEZO1 expression and activation. Under the condition of myelofibrosis, a stiff extracellular matrix impairs megakaryocyte maturation and proplatelet formation by inducing PIEZO1 overexpression. This causes thrombocytopenia and megakaryocytosis, with atypical immature megakaryocytes, which hallmark myelofibrosis.

Overall, this study highlights the involvement of the mechanosensor PIEZO1 in regulating megakaryocyte maturation and proplatelet biogenesis^8^ (Figure 1). Upregulated PIEZO1 in PMF likely contributes to the aberrant megakaryopoiesis associated with this disease and negatively impacts platelet biogenesis. The discovery that PIEZO1 acts as a brake of megakaryocyte differentiation and platelet production opens up the opportunity to develop targeted therapies for megakaryocytes and/or platelet disorders associated with altered bone marrow microenvironment and impaired mechanosensation.

## AUTHOR CONTRIBUTIONS

Francesca Vinchi conceived and wrote the Hematopic.

## CONFLICT OF INTEREST STATEMENT

Dr. Vinchi is a member of the advisory board of Silence Therapeutics and a consultant for RallyBio. None of these relationships is relevant to the current publication.

### FUNDING

Dr. Vinchi receives research funding from CSL Vifor, Silence Therapeutics, and PharmaNutra, none of these relevant for this work.

## Data Availability

Data sharing is not applicable to this article as no datasets were generated or analyzed during the current study.
